# Compressive Properties of Additively Manufactured Metal-Reinforced PLA and ABS Composites

**DOI:** 10.3390/polym16142008

**Published:** 2024-07-13

**Authors:** Meelad Ranaiefar, Mrityunjay Singh, Jonathan A. Salem, Michael C. Halbig

**Affiliations:** 1 NASA Glenn Research Center, Cleveland, OH 44135, USA; jonathan.a.salem@nasa.gov (J.A.S.); michael.c.halbig@nasa.gov (M.C.H.); 2 Ohio Aerospace Institute, Cleveland, OH 44142, USA; mrityunjay.singh-1@nasa.gov

**Keywords:** acrylonitrile butadiene styrene (ABS), additive manufacturing, compression, fused filament fabrication, mechanical properties, metal-reinforced ABS, metal-reinforced polylactic acid (PLA), microstructure, polymer composites

## Abstract

The development of multi-material filaments has enabled fused filament fabrication-based additive manufacturing to address demand for high-performance lightweight multifunctional components. In this study, polylactic acid (PLA) and acrylonitrile butadiene styrene based filaments with metallic reinforcements of magnetic iron (MI), stainless steel (SS), bronze (Br), copper (Cu), Bismuth (Bi), and Tungsten (W) were investigated to elucidate their complex processing–structure–property relationships. The microstructure of 3D-printed materials were characterized by microscopy and analyzed to determine the metal cross-sectional area percentage and the relationship between metal reinforcement, the polymer matrix, and porosity. Compression testing was conducted in directions parallel and perpendicular to the build direction in order to evaluate the effect of orientation and metal reinforcement on the mechanical properties. 3D-printed specimens experienced either fracture through print layers or layer-wise interfacial rupture for loads applied perpendicular and parallel to the print layers, respectively. A dependence of yield strength on loading orientation was observed for Br-PLA, Cu-PLA, SS-PLA, Bi-ABS, and W-ABS; however, MI-PLA and pure ABS specimens did not exhibit this sensitivity. Metal reinforcement also influenced the magnitude of compressive yield strength, with MI-PLA and SS-PLA demonstrating increased strength over Br-PLA and Cu-PLA, while ABS demonstrated increased strength over Bi-ABS and W-ABS. These results demonstrate the importance of considering orientation in printing and applications, the trade-off between various metallic reinforcements for added multifunctionality, and the potential of these tailored polymer composites for novel 3D-printed structures.

## 1. Introduction

In recent years, the demand for lightweight multifunctional components and systems has ignited the exploration into novel materials and manufacturing processes, catalyzing significant advancements across the automotive, aerospace, energy, and medical domains [1,2,3]. Where traditional manufacturing methods, predominantly operated in a subtractive manner, have fallen short, the emergence of additive manufacturing (AM) has precipitated a paradigm shift by enabling the meticulous layer-by-layer construction of objects and delivering an array of advantages compared to conventional techniques [4,5]. In response to this trend, a diverse spectrum of 3D printing systems has evolved to encompass high-end commercial fused filament fabrication (FFF) machines as well as accessible desktop 3D printers. This proliferation of 3D printing technology has propelled the widespread adoption of AM, facilitating the rapid production of components with intricate geometries and without the constraints of intricate tooling [6,7,8].

Amid the spotlight on FFF 3D printing, acrylonitrile butadiene styrene (ABS) and polylactic acid (PLA) emerged as highly compatible and accessible filament materials displaying good print quality and properties [9,10]. Coupled with a growing focus towards augmenting the capabilities of FFF through the incorporation of nanoparticles and other reinforcing agents, both ABS and PLA have become prime contenders as matrices to leverage multifunctional capabilities [11,12]. The integration of metal reinforcements into ABS and PLA matrices holds significant promise for the development of advanced materials with tailored properties, paving the way for components that not only possess mechanical strength, but also exhibit attributes such as thermal conductivity, electrical resistivity, and even magnetic properties. This, in turn, has kindled a keen interest in innovative applications such as Titanium–PLA for orthopedic implants, Copper–PLA for satellite actuators, Magnetic Iron–PLA for transformer cores, and Bismuth–ABS for radiation shielding equipment, exemplifying the remarkable potential of this technology [13,14,15,16]. The addition of harder metallic particles to a softer polymer matrix also promotes wear resistance for potential applications such as bearings and bushings [17].

**Figure 1:**
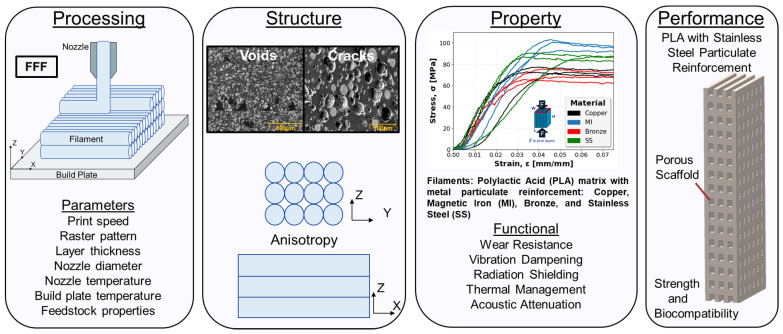
Processing–structure–property–performance diagram for fused filament fabrication (FFF) additive manufacturing.

In order to fully realize the potential of these multi-material components fabricated through FFF, a thorough understanding of process parameters and their corresponding effect on component structure and properties is required [18]. As illustrated in Figure 1, FFF, also referred to as fused deposition modeling (FDM), is a complex process consisting of a multitude of parameters such as print speed, raster pattern, print height, nozzle diameter, nozzle temperature, build plate temperature, and feedstock properties. As parts are built up line by line and layer by layer, the various combinations of these parameters have compounding and significant effects resulting in failed or successful prints. The influence on structure is realized through defects such as voids and cracks or engineered design utilizing lattice structures and porous infill to achieve lightweighting or added functionality [19,20,21]. The influence of structure, as well as material, then has a cascading effect on the component properties including wear resistance, vibrational dampening, radiation shielding, thermal management, and acoustic attenuation. By leveraging the intricate processing–structure–property (PSP) relationship, FFF and novel multi-material systems can be optimized with a tailored multifunctional design to meet increasingly challenging performance requirements.

In addition to the previously discussed parameters, another important aspect influencing the performance of a component is orientation. Due to FFF’s layer-by-layer process, components are inherently anisotropic and demonstrate variation in mechanical strength dependent on the orientation of printed layers relative to an applied load [22,23,24,25]. Fisher et al. [26] demonstrated the influence of orientation on mechanical properties for both nylon reinforced with short carbon fiber and nylon. Although nylon did not demonstrate a change in compressive modulus, the reinforced nylon showed a 130% increase with load applied parallel to the print layers as compared to perpendicular. Emphasizing the complexity and importance of understanding PSP relationships, both orientation and the addition of reinforcement material were shown to influence the mechanical properties of 3D-printed components.

In the current study, both PLA and ABS with various metal reinforcement were printed by FFF. The influence of metal reinforcement particulates and orientation for compressive loading was investigated. The PSP relationship of these materials was evaluated through microstructural analysis and the analysis of mechanical properties including compressive elastic modulus and compressive yield strength. The characterization and understanding of 3D-printed multi-materials ascertained in this research will help inform process optimization and the tailored design of these composites for multifunctional applications and improved mechanical performance.

## 2. Materials and Methods

### 2.1. Materials

The metal-reinforced PLA and ABS filaments and corresponding supplier in this study include bronze-reinforced PLA (colorFabb, Belfeld, Limburg, The Netherlands), copper-reinforced PLA (colorFabb), magnetic iron-reinforced PLA (Proto-pasta, Vancouver, WA, USA), stainless steel-reinforced PLA (Proto-pasta), ABS (3DXTech, Grand Rapids, MI, USA), tungsten-reinforced ABS (GMASS Turner Medtech, Orem, UT, USA), and bismuth-reinforced ABS (GMASS). These materials will hereby be referred to as Br-PLA, Cu-PLA, MI-PLA, SS-PLA, ABS, W-ABS, and Bi-ABS, respectively.

### 2.2. 3D-Printing Process

The filaments were printed on a commercially available Makerbot Replicator 2X (MakerBot Industries, LLC One MetroTech Center, Brooklyn, NY, USA). The print layer height was maintained at a constant 0.3 mm for all materials, and parts were printed with a raster angle alternating between 0° and 45°. The extruder temperature ranged from 200 °C to 220 °C with a bed temperature of 45–60 °C. Coupons of 50 mm length, 20 mm width, and 10 mm height were fabricated for each material.

### 2.3. Microstructural Analysis

Samples were machined from the 3D-printed coupon of each material and mounted using a two-component epoxy resin and hardener (Metlab Corporation, Niagra Falls, NY, USA). The mounted samples were sanded with SiC until plane, and a progressive polishing procedure from 9 µm to 1 µm was applied. Several hours in a colloidal silica concluded the polishing procedure for microstructural analysis. Printed cross-sections were analyzed for all materials by optical microscopy. Constituent elements were identified for each material by scanning electron microscopy (SEM) and energy-dispersive X-ray spectroscopy (EDS). Porosity from the pullout of reinforcement material and measurements for metallic particle area percentage were determined by ImageJ (National Institutes of Health public domain software version 1.8.0, Bethesda, MD, USA) analysis of optical micrographs. This consisted of over 200 individual micrograph images stitched together and analyzed for each material, providing a representative average of pullout porosity and metallic particle area across each 3D-printed cross-section.

### 2.4. Compression Testing

From the remainder of each material’s 3D-printed coupon, 6 specimens, each with approximate dimensions of 10 mm, 6.4 mm, and 10 mm in length, width, and height, respectively, were machined. This resulted in a total of 42 specimens for Br-PLA, Cu-PLA, MI-PLA, SS-PLA, ABS, W-ABS, and Bi-ABS. An Instron 8562 test frame was used to test the specimens under compression according to ASTM D695-15 [27]. Digital image correlation (DIC) was conducted with an ARAMIS (Trilion Quality Systems, Plymouth Meeting, PA, USA) system.

### 2.5. Test Setup and Orientation

**Figure 2:**
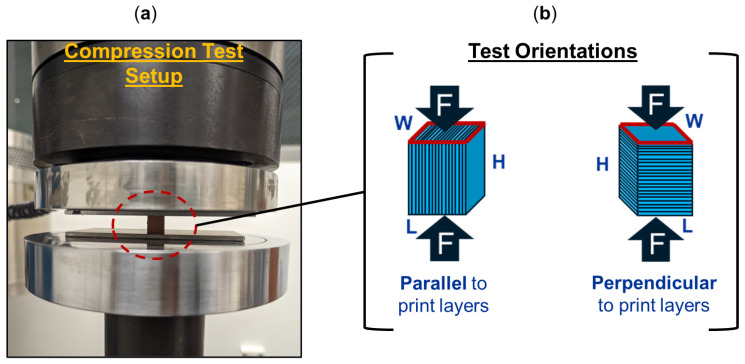
Compression test (**a**) setup and (**b**) representative test orientations of additively manufactured specimens, with compressive force applied either parallel or perpendicular to the print layers.

Figure 2a illustrates the compression test setup and Figure 2b provides a schematic of the two orientations for which the 3D-printed specimens were tested under compression. Three specimens were tested in each orientation, either with a compressive load applied parallel to the print layers or perpendicular to the print layers. The Young’s modulus and compressive yield strength were determined from the test data of each specimen.

## 3. Results and Discussion

### 3.1. Material Characterization—SEM and EDS

**Figure 3:**
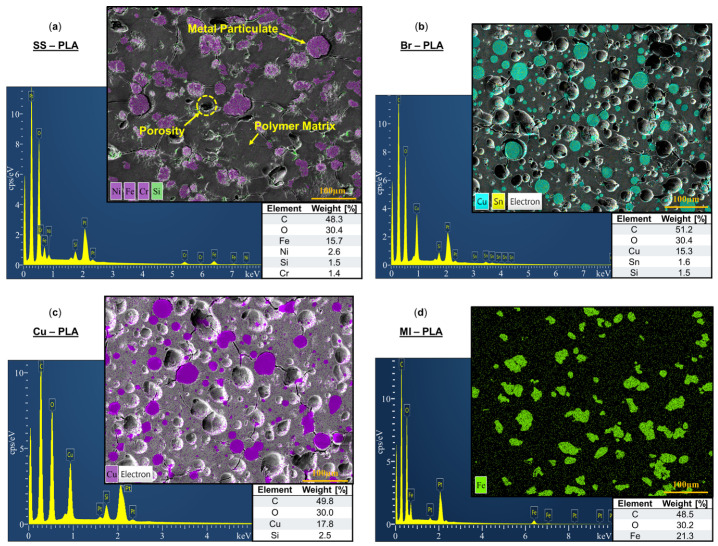
Electron dispersion spectroscopy with weight [%] of constituent elements for (**a**) stainless steel (SS)-PLA; (**b**) bronze (Br)-PLA; (**c**) copper (Cu)-PLA; and (**d**) magnetic iron (MI)-PLA.

SEM and EDS were performed on the cross-section of 3D-printed Br-PLA, Cu-PLA, MI-PLA, SS-PLA, ABS, W-ABS, and Bi-ABS specimens. In Figure 3a–d are SEM micrographs and corresponding EDS spectra for SS-PLA, Br-PLA, Cu-PLA, and MI-PLA, respectively. The constituent elements of the metallic reinforcement for each material are identified in the micrographs.

From Figure 3a, SS-PLA reports a 15.7 weight (wt.) %, 2.6 wt. %, and 1.4 wt. % of iron, nickel, and chromium, respectively. A small amount of impurity, 1.5 wt. % silicon, is also identified, likely a residue from the final polishing step in colloidal silica and not originating from the filament. Closer inspection of the SS-PLA micrograph also reveals pullout porosity, somewhat round and similar in shape to many of the identified metallic particulates. This porosity is likely a consequence of the polishing procedure, where contact of hard metal particles with abrasive sandpaper resulted in the dislodging of a portion of metallic particulates from the cross-sectional surface and matrix of the printed specimen. A thin gap can also be found along the perimeter of several particulates, an early sign of these particulates eroding the surrounding soft PLA matrix as they jostle loose. Additional signs of cracking are observed throughout the matrix, stretching between several metallic reinforcement particulates. Although not detrimental to the performance of components printed with this material, it should be noted that an improved interface between metal particulates and the matrix could result in strengthened mechanical performance. The development of these multi-material filaments is just one more step in the complex PSP relationship requiring a careful balance of binder composition and filler properties for optimized performance [28,29,30].

Figure 3b reports alloying constituents of 15.3 wt. % copper and 1.6 wt. % tin for Br-PLA, Figure 3c reports 17.8 wt % copper for Cu-PLA, and Figure 3d reports 21.3 wt. % iron for MI-PLA. Similar to SS-PLA, EDS spectra capture trace amounts of silica stemming from the final polishing step in colloidal silica. Pullout porosity from the dislodging of reinforcement particulates, as observed with SS-PLA, is also evidenced in the micrographs. Fractures spanning between particulates locked in place and the remaining imprints of those dislodged further characterize an inherent defect when printing with these metal-reinforced filament materials. In addition to previously discussed resolutions such as optimized binder and filler properties, it is possible that the heating and cooling cycle during the 3D printing process combined with a thermal expansion mismatch between the matrix and metallic reinforcement particulates results in the generation of residual stresses and cracking observed in these FFF multi-material specimens. The larger coefficient of thermal expansion of ABS and PLA, relative to their metal reinforcement, results in a greater expansion and contraction of these polymers upon extrusion and cooling, respectively. The differential in contraction between the matrix and reinforcement materials creates internal stresses and the formulation of cracks within the composite. Alternate combinations of particulate size, material, and binder could be explored to identify improved material processing routes. Printing in a closed chamber with a higher and uniform temperature distribution or another form of post-processing could also help alleviate these stresses and reduce crack formation. Furthermore, a slow cooling procedure could allow stresses to be revived through local plastic deformation or creep rather than cracking.

**Figure 4:**
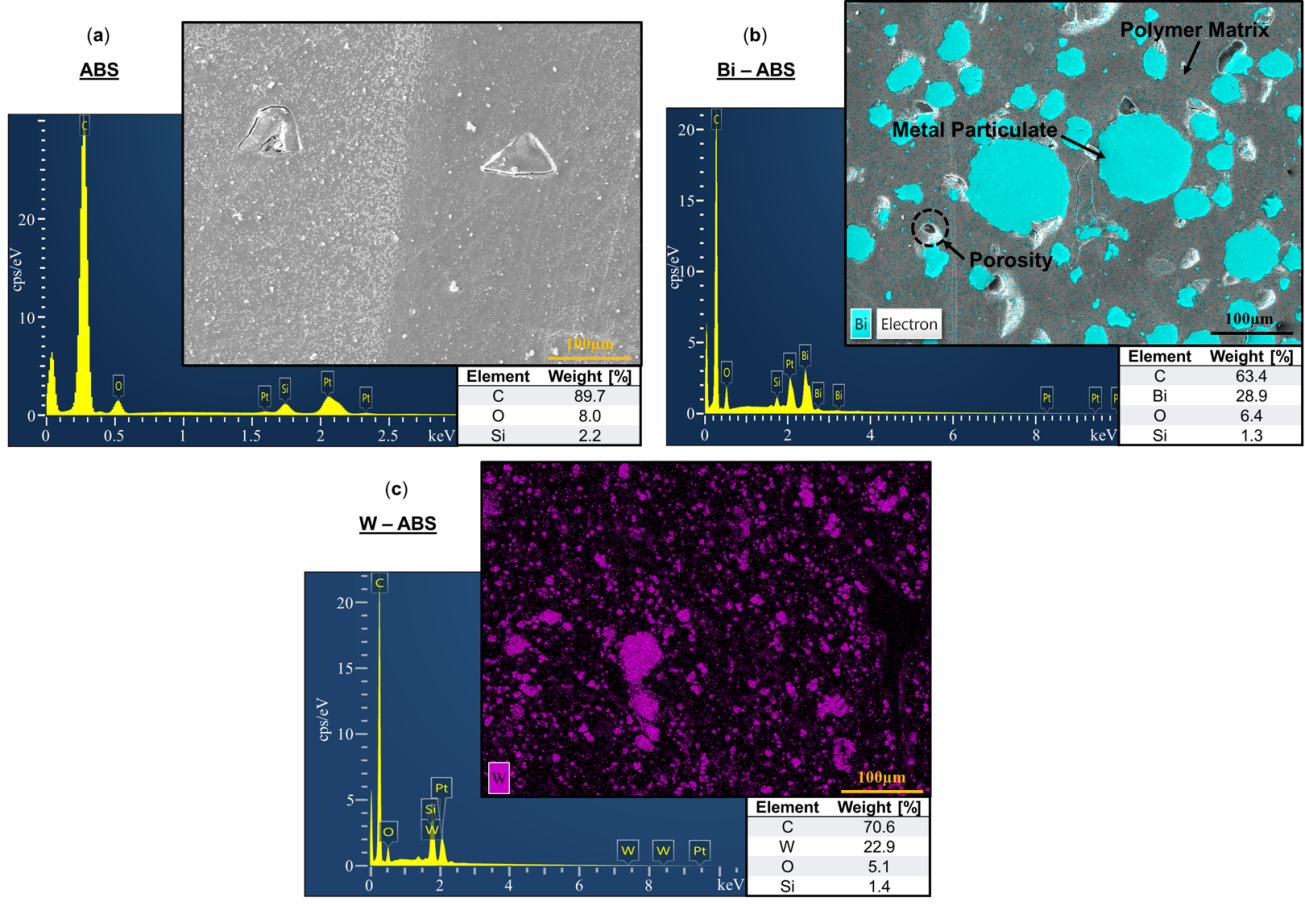
Electron dispersion spectroscopy with weight [%] of constituent elements for (**a**) ABS; (**b**) bismuth (Bi)-ABS; and (**c**) tungsten (W)-ABS.

SEM micrographs and corresponding EDS spectra for ABS, Bi-ABS, and W-ABS are shown in Figure 4a–c, respectively. The constituent elements of the metallic reinforcement for each material are identified in the micrographs. Given that ABS had no metallic reinforcement, Figure 4a EDS results show only 2.2 wt. % silica as a residual impurity from polishing. Figure 4b reports 28.9 wt. % bismuth for Bi-ABS and Figure 4c reports 22.9 wt. % for tungsten for W-ABS; each of the materials have a small amount of residual silica. A wide range of particulate sizes is observed for Bi-ABS and W-ABS and, unlike the metal-reinforced PLA specimens, there are minimal indicators of cracking between particulates. This is possibly due to the better adhesion and balance of filler material and binder in metal-reinforced ABS-based filaments as compared to the metal-reinforced PLA-based filaments. Additional investigation of the interfacial interaction for these multi-materials is warranted. It should be noted that signs of pullout porosity were still identified in Bi-ABS and are a common theme across the polished metal-reinforced ABS and PLA composites.

### 3.2. Material Characterization—Optical Microscopy

**Figure 5:**
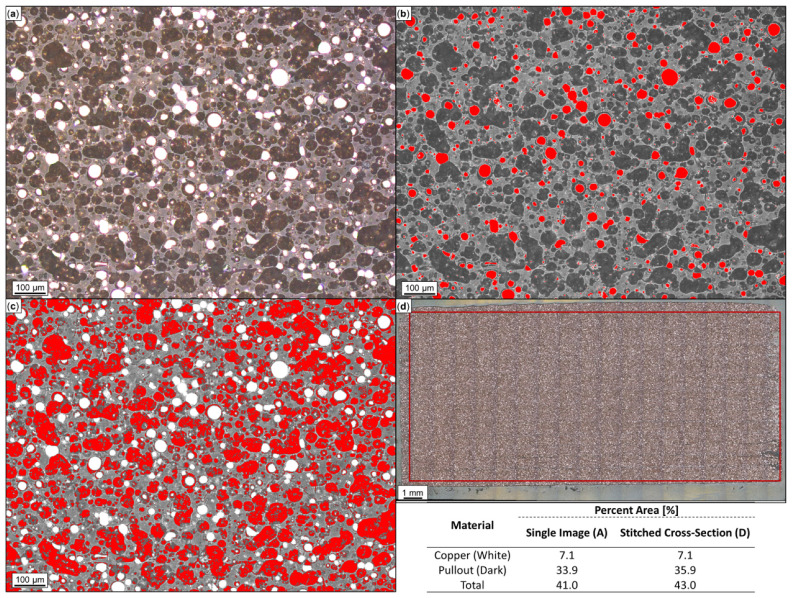
Microscopy and ImageJ porosity quantification of printed copper (Cu)-PLA: (**a**) single optical image; (**b**) copper particles identified in red; (**c**) pullout identified in red; and (**d**) Cu-PLA cross-section comprised of 259 stitched images with analysis constrained to demarcated region. Metal particulates and pullout areas are summed to approximate total area percentage of metal.

Optical microscopy and ImageJ analysis were performed on the cross-section of 3D-printed Br-PLA, Cu-PLA, MI-PLA, SS-PLA, ABS, W-ABS, and Bi-ABS specimens. Shown in Figure 5a–d are optical micrographs of Cu-PLA. In Figure 5a, a single micrograph depicts both white and dark regions. The white regions represent metallic reinforcement; the dark regions are where the pullout of reinforcement material occurred, and the surrounding material is the PLA matrix. These dark regions are believed to be areas of particulate pullout resulting from polishing, as they resemble the shape and size of metal-reinforcement particles still present in the cross-section. For this reason, the area percentage of metallic particulates was determined to be the cumulative sum of these dark and white areas over the micrograph area. Through ImageJ analysis, the approximated metal particles (white) and pullout (dark) are depicted in Figure 5b and Figure 5c, respectively. The total metal area percentage is reported as 41.0%, consisting of 33.9% from pullout and 7.1% from remaining Cu particles. Figure 5d illustrates a complete cross-section of Cu-PLA, comprised of 259 micrographs stitched together. In order to remove background noise, a reduced area, contained within the boundary of the overlaid box, was used for ImageJ analysis. By analyzing the full Cu-PLA cross-section, bias from a single micrograph should be reduced and an averaged representation of the metal area percentage should be obtained. In this case, the total metal area percentage is reported as 43.0%, consisting of 35.9% from pullout and 7.1% from remaining Cu particles. This is fairly similar to the results from a single micrograph, pointing towards a fairly consistent distribution of metal particles and pullout across the entire cross-section. It should be noted that, observed in the SEM and EDS of SS-PLA, Br-PLA, Cu-PLA, and MI-PLA in Figure 3, the erosion of PLA surrounding the reinforcement particles have possibly contributed to an artificially increased pullout area. The formerly present metal particulates are likely smaller than the dark pullout regions captured in the optical micrographs and translate to a slight overestimate in the approximated pullout and overall metal area percentage.

Following a similar approach, cross-sections for Br-PLA, MI-PLA, and SS-PLA were analyzed by ImageJ and are reported alongside Cu-PLA in Table 1. The metal particle, pullout, and cumulative metal area percent for Br-PLA, MI-PLA and SS-PLA is approximated as (22.6%, 7.8%, 30.4%), (7.3%, 8.8%, 16.1%), and (18.7%, 9.8%, 28.5%), respectively. Vakharia et al. [31] reported a similar total metal area percentage for Br-PLA at 32.9%, MI-PLA at 16.1%, and SS-PLA at 27.2%. However, the total metal area percent of Cu particles in Cu-PLA is approximated as 9.8% higher in the current study. Polishing and the corresponding pullout of particles from the microscopy sample likely contributes to enlarged dark regions and an inflated pullout area percent. From the polishing conditions across tested materials, this is most evident with Cu-PLA. In this case, the softness, particle interface, and process parameters for Cu could play a role in the increased pullout area and help explain some of the variation in Cu reinforcement total area between the studies. A larger study of these parameters and cross-sectional analysis could provide further insight into this processing–structure relationship.

Additionally, the total metal reinforcement area of printed materials is compared with metal area percent values observed in filaments by Vakharia et al. [31], shown in Table 1. This difference in quantified metal reinforcement area can be attributed to the FFF process and the corresponding phases of heating and cooling experienced by the filament during extrusion. When heated, decreased viscosity and gravimetric effects result in the migration and dispersion of metal particulates from filament across the printed beads. It may be that the cross-section capturing all printed layers provides a different metal area percentage than a cross-section perpendicular to the print direction. Additionally, PLA does not typically demonstrate shrinking that could be attributed to a reduced plastic area relative to the metal area in printed specimens [32]. It is more likely that the printing process aided adhesion between metal reinforcement particles and the PLA matrix. The magnitude of this benefit could vary based on the complex interaction of multiple factors including material, surface treatment of the metal particles, print temperature, and cooling rate [33].

**Figure 6:**
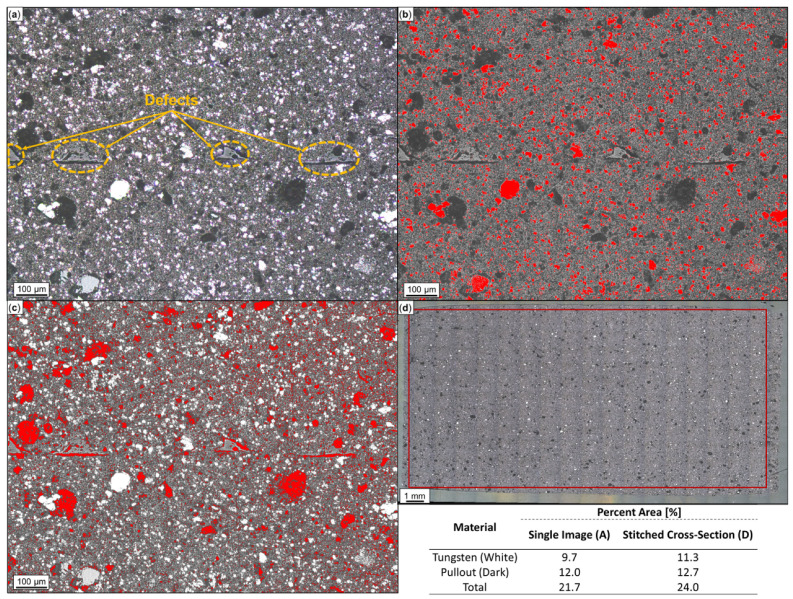
Microscopy and ImageJ porosity quantification of printed tungsten (W)-ABS: (**a**) single optical image; (**b**) tungsten particles identified in red; (**c**) pullout identified in red; and (**d**) W-ABS cross-section comprised of 247 stitched images with analysis constrained to demarcated region. W and pullout areas are summed to approximate total area percent.

In Figure 6a, an optical micrograph from the cross-section of W-ABS is provided. Similar to the metal-reinforced PLA material, white and dark regions represent metal particles and the pullout of metal particles, respectively, while the surrounding material is the ABS matrix. Through ImageJ analysis, the white and dark regions are identified and quantified in Figure 6b at 9.7% and Figure 6c at 12.0%, respectively. The summed total metal area percent, 21.7%, from the single micrograph is fairly similar to the approximated value of 24.0%, generated from the 247 stitched optical micrographs illustrated in Figure 6d. Following a similar procedure and reported in Table 2, Bi-ABS has a metal area of 15.9%, a pullout area of 13.4%, and a total metal reinforcement area of 29.3%. As confirmed by Figure 4a, the plain ABS material does not contain reinforcement material.

### 3.3. Material Characterization—Fracture and Failure

**Figure 7:**
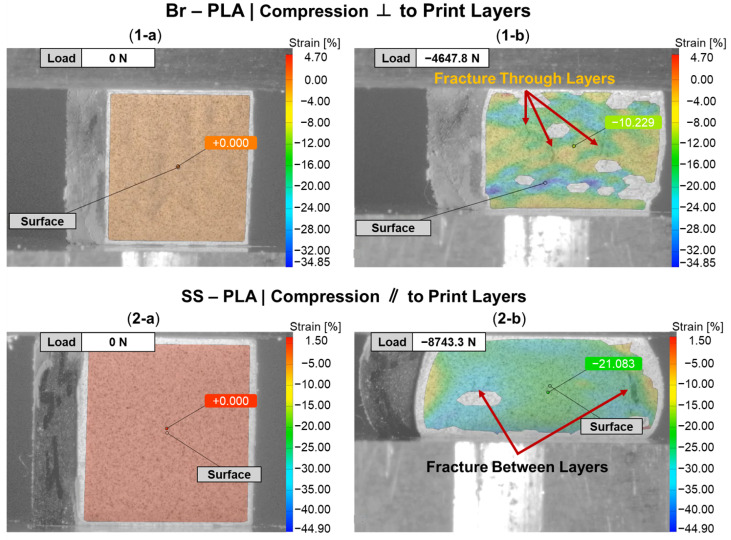
DIC snapshots of subset of PLA specimens during compression testing: (**1**) bronze (Br)-PLA compressed perpendicular (⊥) to print layers; and (**2**) stainless steel (SS)-PLA compressed parallel (‖) to print layers; (**a**) before compression; and (**b**) after compression.

**Figure 8:**
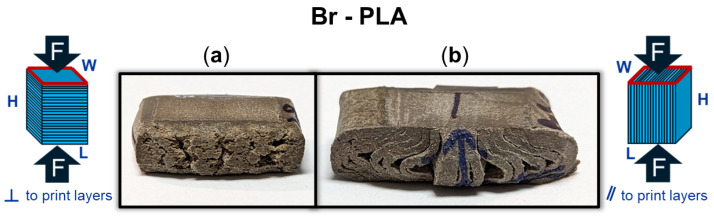
Bronze (Br)-PLA specimen after compression (**a**) perpendicular (⊥) to print layers with stretching and fracture across the layers; and (**b**) parallel (‖) to print layers with delamination of the layers.

Beyond an understanding of the metal reinforcement within the matrix, the orientation of a 3D-printed component can dictate its exhibited mechanical properties. Figure 7 provides a DIC snapshot of a Br-PLA and SS-PLA specimen during compression testing, with a minimal applied load and after yielding. The Br-PLA specimen, Figure 7(1-a,1-b), had a compressive load applied perpendicular to the print layers. Upon yielding, the specimen demonstrated the initial stages of intra-layer rupture across multiple print layers. Conversely, the SS-PLA specimen, in Figure 7(2-a,2-b), had a compressive load applied parallel to the print layers. In this case, upon yielding, the specimen demonstrates signs of delamination. Given that the interfacial bond between layers is a point of weakness, prone to porosity, the inter-layer fracture observed with load applied parallel to the print layers is expected. Figure 6a captures several of these porosity defects, in the shape of triangles, occurring at the junction of two lines within the the same layer and the the surface of the preceding layer. These junctions contribute to a reduced contact area and adhesion between layers, likely serving as initiation points for any fracture and propagation that occurs under loading conditions. When load is applied perpendicular to the print direction, these junctions and the inter-layer adhesion do not play as significant of a role because the defects are compressed closed. If the loading was in tension, then the defect would substantially weaken the material. Fractures mainly propagate vertically through the layers, secondarily spreading in the horizontal direction across the layer-to-layer interface. This horizontal propagation could have also been mitigated in part due to the compressive load collapsing any existing porosity and removing these structural weak points as propagation pathways. A post-compression test specimen of Br-PLA loaded perpendicular and parallel to the print layers is captured in Figure 8a and Figure 8b, respectively. These fracture patterns were observed consistently across the metal-reinforced PLA specimens of Br-PLA, Cu-PLA, MI-PLA, and SS-PLA.

**Figure 9:**
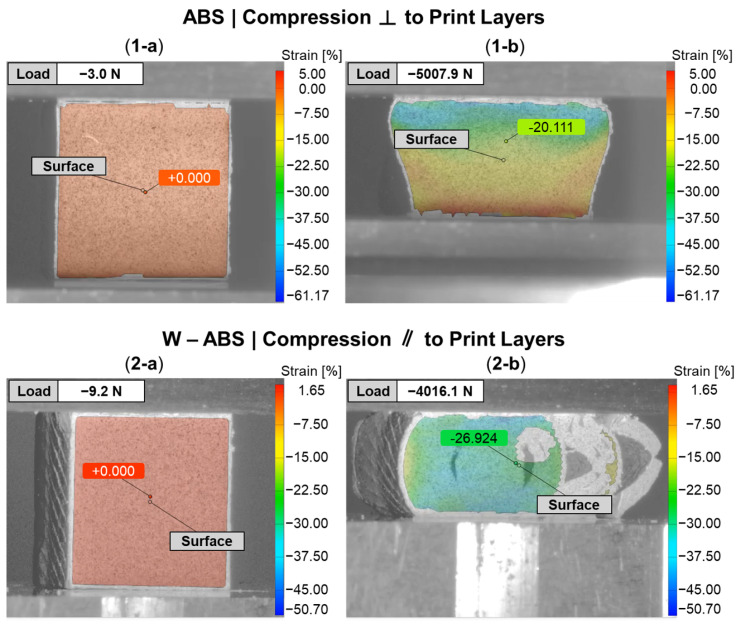
DIC snapshots of subset of ABS specimens during compression testing: (**1**) ABS compressed perpendicular (⊥) to print layers; and (**2**) tungsten (W)-ABS compressed parallel (‖) to print layers; (**a**) before compression; and (**b**) after compression.

**Figure 10:**
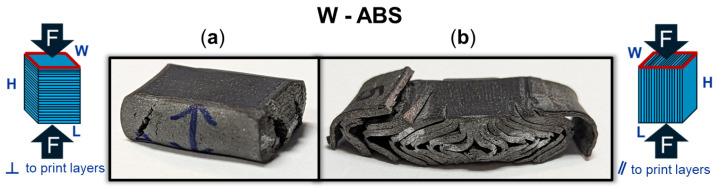
Tungsten (W)-ABS specimen after compression (**a**) perpendicular (⊥) to print layers with stretching and fracture across the layers; and (**b**) parallel (‖) to print layers with delamination of the layers.

Figure 9 provides a DIC snapshot of an ABS and W-ABS specimen during compression testing, with a minimal applied load and after yielding. A compressive load was applied perpendicular to the print layers for the ABS specimen in Figure 9(1-a,1-b). Similar to the metal-reinforced PLA specimens loaded perpendicular to the print layers, interfacial adhesion and junction point porosity do not play a significant role in the its failure. However, the ABS specimen also does not display signs of rupture through the print layers. It is possible that the ABS specimen was not as brittle as its metal-reinforced counterparts, allowing it to maintain a degree of malleability and conform to the applied compressive load. This was the case for all three ABS specimens loaded perpendicular to the print layers. In Figure 9(2-a,2-b), W-ABS is loaded in compression parallel to the print layers. As observed with the metal-reinforced PLA specimens, a fracture occurs along the interfacial boundary at multiple sites. This is, again, due to the porosity located at the junction of adjacent layers providing an initiation point for fractures and propagation to occur. However, unlike other specimens loaded parallel to the print direction, W-ABS failure results in significant separation between each layer, with outer layers completely separating from the remainder of the specimen. This was consistent for all W-ABS specimens loaded parallel to the print layers and is possibly a consequence of poor inter-layer adhesion stemming from processing conditions along with brittle material behavior. When loaded perpendicular to the print layers, the W-ABS specimens fails by rupture through the print layers, similar to the metal-reinforced PLA specimens. A post-compression test specimen of W-ABS loaded perpendicular and parallel to the print layers is captured in Figure 10a and Figure 10b, respectively. Unlike ABS loaded perpendicular to the print layers and W-ABS loaded parallel to the print layers, Bi-ABS exhibited failure similar to the metal-reinforced PLA specimens in both orientations. The differences in failure response of these materials under compression is indicative of the underlying complex structure–property relationship, where the effect of process parameters, porosity, and interfacial strength on mechanical strength is warranted.

### 3.4. Mechanical Properties—Stress–Strain Curves

**Figure 11:**
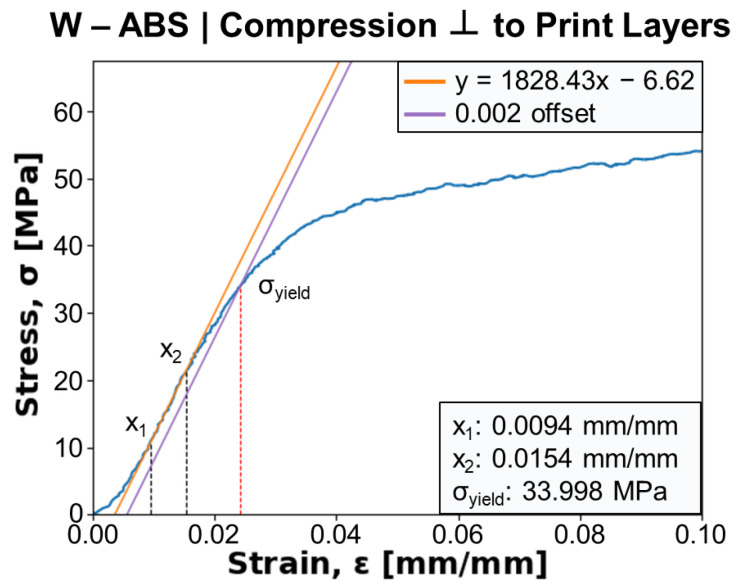
Stress–strain curve, denoted in blue, for a tungsten (W)-ABS specimen compressed perpendicular to print layers. Young’s modulus is calculated from x_1_ and x_2_ bounds, and the yield strength, $\sigma_{yield}$, is determined from the 0.002 offset intersection.

Figure 11 shows a representative stress–strain curve from the compression testing of a W-ABS specimen with force applied perpendicular to the print layers. The Young’s modulus, *E*, is determined from the slope between two points, $x_{1}$ and $x_{2}$, in the linear elastic region of the mechanical response. A 0.2% offset parallel to the Young’s modulus line is generated and its intersection with the stress–strain curve is taken as the compressive yield strength, $\sigma_{yield}$, of the specimen. For the current specimen, *E* and $\sigma_{yield}$ are calculated as approximately 1828 MPa and 34.0 MPa, respectively. A similar procedure was used to calculate these values for all metal-reinforced PLA and ABS specimens.

**Figure 12:**
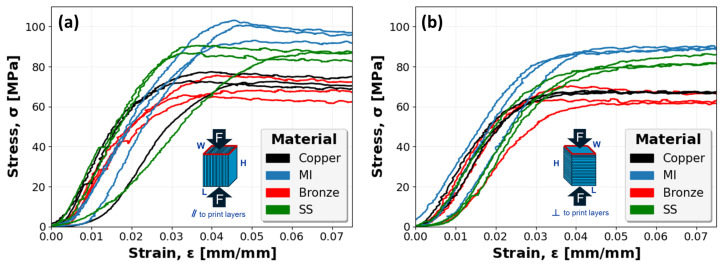
Stress–strain curves for PLA specimens reinforced with copper (Cu), magnetic iron (MI), bronze (Br), or stainless steel (SS): (**a**) specimens compressed parallel (‖) to print layers; and (**b**) specimens compressed perpendicular (⊥) to print layers.

Figure 12 captures the stress–strain mechanical response of all Cu-PLA, MI-PLA, Br-PLA, and SS-PLA specimens. The results for specimens with print layers oriented parallel with the compressive force, in Figure 12a, are generally consistent for each material. However, a single specimen of each Cu-PLA and SS-PLA capture a slight deviation in the linear elastic region relative to other specimens. This is likely a result of print defects and associated variability in specimen structure requiring more initial displacement to compress and collapse the material. However, after some compression, these specimens eventually attain stress values similar to their material counterparts. The stress–strain curves for specimens with print layers oriented perpendicular to the compressive force are shown in Figure 12b. These specimens follow a similar trend as those compressed in the parallel direction, although slightly weaker, demonstrating a general consistency in the response of each material group.

**Figure 13:**
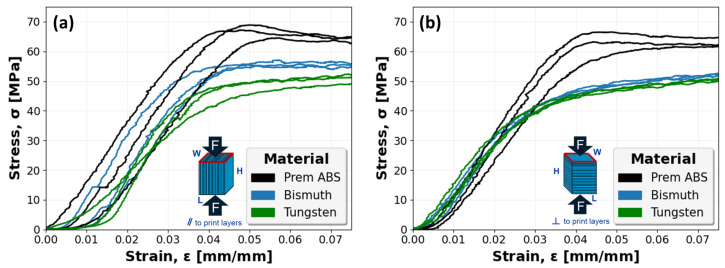
Stress–strain curves for ABS, bismuth (Bi)-ABS, and tungsten (W)-ABS specimens: (**a**) specimens compressed parallel (‖) to print layers; and (**b**) specimens compressed perpendicular (⊥) to print layers.

Similarly, Figure 13 captures the stress–strain mechanical response of all ABS, Bi-ABS, and W-ABS specimens. Specimens with a compressive load applied parallel to the print layers and perpendicular to the print layers are shown in Figure 13a and Figure 13b, respectively. In both orientations, a fairly consistent compressive response is observed.

### 3.5. Mechanical Properties—Young’s Modulus and Yield Strength

**Figure 14:**
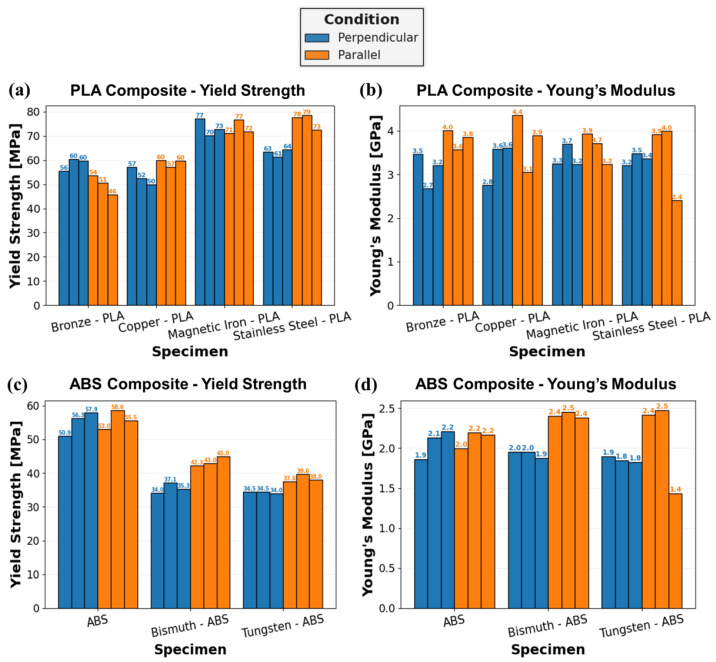
Bar chart of compression test results for metal-reinforced PLA and ABS specimens: (**a**) yield strength of PLA specimens; (**b**) Young’s modulus of PLA specimens; (**c**) yield strength of ABS specimens; and (**d**) Young’s modulus of ABS specimens. Orientation of specimen compression relative to print layers is color coded: Blue—perpendicular; and Orange—parallel.

The yield strength and Young’s modulus of the metal-reinforced PLA and ABS specimens, with a compressive load applied either perpendicular or parallel to the print layers, is quantified in Figure 14. The yield strength of Br-PLA, Cu-PLA, MI-PLA, and SS-PLA, in Figure 14a, shows a distinct difference in response according to the metal reinforcement. Both MI-PLA and SS-PLA reach larger yield strength values than Br-PLA and Cu-PLA, in both test orientations. When a compressive load is applied perpendicular to the print layers, Br-PLA exhibits an increased average yield strength, 58.7 MPa, relative to the parallel loading direction, 50.3 MPa. Conversely, Cu-PLA and SS-PLA exhibit an increased average yield strength when a compressive load is applied parallel to the print layers, 59 MPa and 76.7 MPa, respectively, compared to the perpendicular orientation, 53 MPa and 62.7 MPa, respectively. A similar average yield strength of 73.3 MPa was observed in both loading orientations for MI-PLA. Not only does this emphasize the influence of metal reinforcement on PSP relationships, but it demonstrates how the effect of loading relative to the orientation of print layers varies between materials.

A connection may also be made with the total area percent of metal reinforcement observed within each composite. The ranked order of composites from least to largest quantified total reinforcement area is MI-PLA, SS-PLA, Br-PLA, and, finally, Cu-PLA. This order matches the ranking of largest to smallest compressive yield strength. Extrapolating from this trend, it is hypothesized that a PLA specimen with no metal reinforcement would exhibit the largest compressive strength of these materials, as observed in a separate study comparing compressive properties of PLA and MI-PLA [34]. This trend of decreasing yield strength with increasing percentage of metal reinforcement was also observed by Vakharia et al. [31] in the tensile testing of PLA, Br-PLA, Cu-PLA, MI-PLA, and SS-PLA. This trend is likely, in part, a result of defects and interfacial interactions stemming from the introduction of metal particles into the PLA matrix.

In Figure 14b, the Young’s modulus of metal-reinforced PLA specimens is quantified. Unlike yield strength, the average Young’s modulus is larger for all Br-PLA, Cu-PLA, MI-PLA, and SS-PLA specimens with a compressive load applied parallel to the print layers rather than in the perpendicular orientation. When loading is in the perpendicular orientation, this results in the closing of pancake defects and a lower modulus. The average Young’s modulus for these materials in the parallel direction is 3.8 GPa, 3.8 GPa, 3.6 GPa, and 3.4 GPa, respectively, compared to values of 3.1 GPa, 3.3 GPa, 3.4 GPa, and 3.4 GPa, respectively, in the perpendicular direction. When the outlier for SS-PLA in the parallel direction is removed, the average Young’s modulus is 4.0 GPa and holds to the trend observed with Br-PLA, Cu-PLA, and MI-PLA. It is noted that pore and defect shape anisotropy affects modulus, where a larger percentage of the compressed area is a void in the perpendicular orientation.

In Figure 14c, the yield strength of ABS, Bi-ABS, and W-ABS is shown. Although the average yield strength for ABS loaded in compression either parallel or perpendicular to the print layers is similar at 55.7 MPa and 55.0 MPa, respectively, this is not the case for the metal-reinforced ABS specimens. Both Bi-ABS and W-ABS exhibit greater average yield strength when loaded parallel to the print layers, 43.4 MPa and 38.4 MPa, respectively, compared to the perpendicular orientation, 35.5 MPa and 34.3 MPa, respectively. It is also observed that ABS has a larger average yield strength than the metal-reinforced counterparts, with Bi-ABS exhibiting a slightly higher strength than W-ABS. From Table 2, Bi-ABS has a greater total area percentage of reinforcement than W-ABS, and does not fit the trend of increasing yield strength with decreased total reinforcement area as found with metal-reinforced PLA specimens. This could be due to differences in the ABS and PLA material system and their interaction with the metal reinforcement particulates. This could be further investigated by testing various concentrations of metal reinforcement to discern the impact on mechanical strength.

The Young’s modulus of ABS, Bi-ABS, and W-ABS is presented in Figure 14d. In this case, ABS exhibits an average Young’s modulus of approximately 2.1 MPa when loaded either parallel or perpendicular to the print layers. However, both Bi-ABS and W-ABS exhibit a greater dependence on orientation. With compressive loading applied parallel to the print direction, Bi-ABS and W-ABS yield an average Young’s modulus of 2.4 GPa and 2.1 GPa, respectively, while loading perpendicular to the print layers results in reduced average values of 2.0 GPa and 1.8 GPa, respectively. If the outlier is removed from W-ABS in the parallel orientation, the average value is approximately 2.5 GPa, significantly greater than in the perpendicular direction. Similar to the metal-reinforced PLA specimens, these metal-reinforced ABS specimen exhibit an increased Young’s modulus when compressively loaded parallel rather than perpendicular to the print layers.

Provided that the pure ABS specimens performed similarly in both orientations, Young’s modulus demonstrates a dependence on metal reinforcement. It is hypothesized that the metal particulates branch between adjacent layers and provide enhanced interfacial adhesion, where the benefit is realized when load is applied parallel to the print layers. This is possible due to the enhanced thermal properties of the metal reinforcement. With an increased density and increased heat retention relative to the matrix material, some metal particulates in an extruded bead path could sink to the bottom and penetrate the interface with the previous layer. Additionally, the geometry of specimens is such that, although they have the same dimensions, those compressively loaded perpendicular to the print layers are comprised of a larger number of layers across the 10 mm height. In specimens tested in the parallel orientation, the number of layers is contained within 6.4 mm. This translates to approximately 12 additional layers and additional porosity which could contribute to a reduced stiffness of specimens loaded perpendicular to the print direction. Although the junction porosity still serves as a prominent crack propagation pathway in the parallel loading orientation, the metal reinforcement, enhanced interfacial adhesion, and reduced number of layers with interfacial porosity provide these specimens with greater stiffness than those loaded perpendicular to the print layers.

## 4. Conclusions

Three-dimensional-printed metal-reinforced specimens of Br-PLA, Cu-PLA, MI-PLA, SS-PLA, Bi-ABS, W-ABS, and ABS have been evaluated to investigate PSP relationships. From the microscopy of their cross-sections, these 3D-printed materials demonstrated variation in the observed metal reinforcement, pullout, and total area percent. The increased propensity of pullout for certain materials such as Cu-PLA, and general pullout observed in all reinforced composites, demonstrate potentially weak interfacial adhesion between metal particle reinforcement and the matrix. Also of note is the reduced metal area percent observed in filament relative to 3D-printed specimens. This is likely due to beneficial interactions between metal reinforcement, the matrix material, and heating and cooling phases of the extrusion process resulting in improved adhesion relative to that of the filament. Also observed in the metal-reinforced PLA and ABS composites were inherent print defects including fractures propagating between regions of metal particulates, pullout, and voids at the junction of adjacent layer beads. One avenue to resolve these detriments requires further exploration of the processing space to determine optimal parameters such as nozzle temperature, bed temperature, layer height, print speed, raster pattern, and particle surface roughness. Another consideration would require the binders and filler material used in the filament formulation to be optimized. By mitigating voids and strengthening the interface between metal particulates and the matrix for all composite specimens, improved mechanical performance of these 3D-printed components could be achieved.

From compression test results, it was demonstrated that metal reinforcement and orientation had varied effects on yield strength, Young’s modulus, and failure mode. When compressed parallel to the print layers, junction points of porosity served as vertical crack propagation pathways for the delamination of layers. Due to the anistropic nature of 3D printing, junction point voids are pancaked closed when loaded in the perpendicular direction. Significant fracture occurred vertically through the layers and, to a lesser extent, propagated horizontally through the compressed voids. This also contributed to a reduced Young’s modulus in the perpendicular relative to parallel loading orientation. All specimens except for ABS demonstrated an increased average value in the parallel orientation. It is possible that metal reinforcement helped bridge and enhance the adhesion between adjacent layers, resulting in increased stiffness when loaded parallel to the print layers. Also a contributing factor is the reduced number of layers comprising specimens tested in the parallel direction, possibly corresponding to reduced porosity. As for yield strength, Cu-PLA, SS-PLA, Bi-ABS, and W-ABS experienced an increase in average yield strength when compressed in the parallel direction, Br-PLA experienced the converse, and MI-PLA and ABS exhibited similar average values in both orientations. Unlike the metal-reinforced ABS specimens, the metal-reinforced PLA specimens also demonstrated a trend of increasing yield strength with decreasing total reinforcement area percent. Given that these are different material systems, this is not completely unexpected and can be traced back to the interfacial strength between particles and the matrix determined by a culmination of print parameters, powder characteristics, binder material, the matrix material, and the metal reinforcement. Additionally, increased porosity with increasing metal reinforcement percentage could contribute to a reduced yield strength relative to non-reinforced material. By eliminating voids and improving the adhesion between metal reinforcement and the matrix, through previously described methods, both the yield strength and Young’s modulus could be increased.

Although there are avenues for improvement such as with the interface strength of reinforcement particles and the matrix, these results have demonstrated the potential to use metal-reinforced PLA and ABS composites in the design and fabrication of novel 3D-printed components. Additional work in characterizing and evaluating the multifunctionality of these materials through thermal, electrical, magnetic, and mechanical testing is also necessary to develop further understanding of their PSP relationships. Extended work could explore the effect of various metal infill percentages on PSP relationships as well. This information may then be used to guide and inform the design of these multi-material metal-reinforced composites for tailored and optimized applications.

## Figures and Tables

**Table 1:** Measured area percent of metal reinforcement in bronze (Br)-PLA, copper (Cu)-PLA, magnetic iron (MI)-PLA, and stainless steel (SS)-PLA printed specimens. Metal area percent of filaments and printed specimens by Vakharia et al. are included for comparison [31].

Material	Metal Area [%] (Printed)	Pullout Area [%] (Printed)	Total Area [%]		Metal Area [%] (Filament)
			**Current**	**Other**	
Br-PLA	22.6	7.8	30.4	32.9	13.9
Cu-PLA	7.1	35.9	43.0	33.2	3.2
MI-PLA	7.3	8.8	16.1	16.1	3.4
SS-PLA	18.7	9.8	28.5	27.2	19.4

**Table 2:** Measured area percent of metal reinforcement in ABS, bismuth (Bi)-ABS, and tungsten (W)-ABS printed specimens.

Material	Metal Area [%]	Pullout Area [%]	Total Area [%]
ABS	-	-	-
Bi-ABS	15.9	13.4	29.3
W-ABS	11.3	12.7	24.0

## Data Availability

The raw data supporting the conclusions of this article will be made available by the authors on request.

## Abbreviations

The following abbreviations are used in this manuscript:

ABS	Acrylonitrile butadiene styrene
AM	Additive manufacturing
Bi	Bismuth
Br	Bronze
Cu	Copper
EDS	Energy dispersive X-ray spectroscopy
DIC	Digital image correlation
FDM	Fused deposition modeling
FFF	Fused filament fabrication
MI	Magnetic iron
PLA	Polylactic acid
PSP	Processing–structure–property
SEM	Scanning electron microscope
SS	Stainless steel
W	Tungsten
